# The microstructure of Si surface layers after plasma-immersion He^+^ ion implantation and subsequent thermal annealing^1^

**DOI:** 10.1107/S1600576717003259

**Published:** 2017-03-22

**Authors:** Andrey Lomov, Kirill Shcherbachev, Yurii Chesnokov, Dmitry Kiselev

**Affiliations:** a Institute of Physics and Technology, Russian Academy of Sciences, Moscow, Russian Federation; b National University of Science and Technology (MISiS), Moscow, Russian Federation; c Institute of Microelectronics Technology, Chernogolovka, Russian Federation

**Keywords:** silicon, helium-filled bubbles, plasma-immersion ion implantation

## Abstract

The structural changes in the surface layer of p-type Cz-Si(001) samples after high-dose low-energy (2 keV) He^+^ plasma-immersion ion implantation and subsequent thermal annealing were studied using a set of complementary methods: high-resolution X-ray reflectometry, high-resolution X-ray diffraction, transmission electron microscopy and atomic force microscopy. The formation of a three-layer structure was observed (an amorphous a-SiO_*x*_ layer at the surface, an amorphous a-Si layer and a heavily damaged tensile-strained crystalline c-Si layer), which remained after annealing. Helium-filled bubbles were observed in the as-implanted sample. The influence of annealing on the evolution of the three-layer structure and the bubbles is considered. The bubbles are shown to grow after annealing. Their characteristic size is determined to be in the range of 5–20 nm. Large helium-filled bubbles are located in the amorphous a-Si layer. Small bubbles form inside the damaged crystalline Si layer. These bubbles are a major source of tensile strain in the c-Si layer.

## Abstract 2

A set of complementary methods (high-resolution X-ray reflectivity, high-resolution X-ray diffraction, transmission electron microscopy and atomic force microscopy) were used to study structural changes in the surface Si layer after high-dose low-energy (2 keV) He^+^ plasma-immersion ion implantation and subsequent thermal annealing. This combination is proved to be a powerful tool for complete structural diagnostics of nanoscale He^+^ ion implanted Si layers, especially in the ultra-low-energy implantation regime.

## 1. Introduction   

Over the past decade (Wesch & Wendler, 2016 ▸), the implantation of noble gas ions has attracted growing interest because of the formation of nanocavities (voids and gas-filled bubbles). As is well known (Nastasi & Mayer, 2006 ▸), light ions are considered preferable because of their low surface sputtering effect. Subsequent research showed that these voids have clean inner surfaces (dangling bonds) that can trap metallic impurities (Follstaedt *et al.*, 1996 ▸). To control these unwanted impurities, gettering treatment is required, and cavities have been shown to be a powerful technique to locally getter metallic impurities (Roqueta *et al.*, 2000 ▸). The defects induced by cavity formation can be used for very effective strain relaxation in pseudomorphic SiGe/Si heterostructures (Trinkaus *et al.*, 2000 ▸). Gas-filled bubbles created by a combination of high-dose He and H implantation are also applied in the ‘Smart Cut’ process as a cutting tool for the production of silicon-on-insulator structures (Bruel, 1996 ▸).

In particular, nanosized cavities in silicon are usually generated by high-fluence He ion implantation at ambient temperature. Owing to its low solubility, He segregates in gas-vacancy complexes and, depending on implantation parameters such as energy and fluence, forms highly pressurized helium-filled bubbles of a few nanometres in size (Chen *et al.*, 1999 ▸). During subsequent annealing at above 973 K, the bubbles grow and He is released from the bubbles because of its ability to permeate from inside the bubble to the matrix, leading ultimately to the formation of empty cavities (voids) (Griffioen *et al.*, 1987 ▸; Raineri *et al.*, 1995 ▸; Seager *et al.*, 1994 ▸).

The formation of helium-filled bubbles can be achieved by a new doping process – high-dose plasma-immersion ion implantation (PIII) (Gupta, 2011 ▸). The high-dose low-energy PIII process has became popular as a cheaper alternative to conventional ion implantation for ion beam synthesis. The method allows one to carry out high-dose implantation over the entire substrate surface with ion energies of 0.5–5 keV. Despite the unique features that make PIII a promising technique, it also has some drawbacks: (1) accurate *in situ* dose monitoring is complicated; (2) the implant energy distribution is inhomogeneous; (3) an ion channelling effect occurs and prevents the formation of sharp interfaces during the synthesis of p–n junctions in Si substrates. However, the formation of bubbles and voids and their further evolution during thermal processing are associated with a transformation of the crystalline microstructure of the damaged layers that changes the electronic properties of the material.

Thus, attaining a deeper insight into the damage accumulation processes in Si and the helium-filled bubble formation mechanisms after high-dose low-energy (<5 keV) He^+^ PIII requires additional investigations. The formation of bubbles affects the point defect fluxes in the matrix and therefore leads to the formation of extended defects with quite distinct morphologies (dislocation loops, rod-like and ribbon-like defects) compared to those occurring in the absence of cavity formation. The final defect morphology resulting from He implantation and annealing is quite complex and may include point and extended defects of vacancy or interstitial type. Factors influencing the final defect morphology are, for example, the damage rate, the thermal and radiation-enhanced mobility of He atoms and point defects, and, in particular, the ability of helium to permeate from the cavities back into the matrix. As an effective sink for radiation-induced defects and He atoms, the physical surface is another important factor, especially for low-energy implantation. As a result, a multilayer structure consisting of sublayers of the material in various states (amorphous, porous, damaged crystalline) forms near the silicon substrate surface after PIII. The fundamental properties of the multilayer structures and the evolution and defect formation mechanism cannot be understood without a detailed investigation of their structural features after thermal annealing.

Study of the microstructure of Si surface layer transformations requires complementary methods. The early stages of bubble formation in 20 keV helium-implanted (001) silicon were studied using Rutherford backscattering spectrometry in channelling conditions, cross sectional transmission electron microscopy and grazing-incidence small-angle X-ray scattering on a synchrotron X-ray source (Pivac *et al.*, 2003 ▸).

Recently, it was shown (Lomov *et al.*, 2014 ▸) that high-resolution X-ray reflectometry (HRXRR) on a laboratory diffractometer can be successfully used for diagnostics of the surface layers in silicon substrates after low-energy (2–5 keV) He^+^ PIII. The layers were shown to have a complex structure which included an amorphized layer, a layer containing encapsulated He-filled bubbles and an elastically strained damaged crystalline layer. For the case of implantation with an energy of 5 keV, it was found that an internal porous layer containing pores of 5–20 nm in diameter forms in the surface region at implantation doses of above 5 × 10^16^ cm^−2^. The layer parameters obtained from the Xray diffraction data were in good agreement with the calculated distribution of implanted helium concentration, and the presence of bubbles and their size were consistent with the transmission electron microscopy (TEM) results. Here we present a report on the microstructure evolution in Si surface layers after high-dose low-energy (2 keV) He^+^ PIII and subsequent annealing, studied using HRXRR, high-resolution X-ray diffraction (HRXRD), TEM and atomic force microscopy (AFM) methods.

## 2. Experiments   

Samples of 30 × 40 mm in size were cleaved out from a p-type (ρ = 12 Ω cm) Cz-Si(001) wafer. High-dose (*D* = 5 × 10^17^ cm^−2^) low-energy (*E* = 2 keV) He^+^ ions were implanted at room temperature in a plasma-immersion low-voltage ion implanter equipped with an inductively coupled plasma source. The target temperature during the process did not exceed 373 K. After the implantation the samples were vacuum annealed at 853 and 1073 K for 30 min.

HRXRD and HRXRR measurements were performed on a multipurpose SmartLab (Rigaku Corporation) diffractometer equipped with a 9 kW copper rotating anode. A high-resolution experiment was set up using a Goebel mirror with a fourfold Ge(220) Bartels-type primary beam monochromator and a twofold Ge(220) diffracted beam analyzer. For this configuration the wavelength dispersion of the primary beam was Δλ/λ ≃ 7 × 10^−5^ and the divergence of the primary and the diffracted beams was only 12′′. X-ray reflectometry measurements were performed in a high-resolution configuration with a beam size of 0.03 mm and a same-sized slit in front of the detector.

To get more detailed information about the local structure of the damaged layer and the He-filled bubbles, the samples were investigated using TEM/STEM (FEI, USA) with a spherical aberration (Cs probe) corrector at an accelerating voltage of 300 kV. The microscope was equipped with a field emission cathode (Schottky), a SuperTwin objective lens with a spherical aberration coefficient of 1.2 mm, an energy dispersion X-ray spectrometer (EDAX, USA), a high-angle annular dark-field electron detector (Fischione, USA) and a Gatan imaging filter (Gatan, Germany). The cross-section samples for TEM were prepared with a focused ion beam of Ga^+^ in a Helios NanoLab 600i scanning electron–ion microscope (FEI, USA) equipped with Pt and W gas injection systems and an Omniprobe 200 micromanipulator (Omni­probe, USA). *Digital Micrograph* (Gatan, USA) and *TIA* (FEI, USA) software were used for image processing. To map the Si layers, we set the 2 eV wide energy window in accordance with the Si plasmon loss (17 eV). Conversely, to obtain a chemical map of the oxide (SiO_2_), we set the energy window at 23 eV. Single-axis tilt series of bright-field TEM images at defocus values of −0.5 µm were collected from −60 to +60° with a tilt angle increment of 2°. The tilt series were aligned and tomograms were reconstructed (by the simultaneous iterative reconstruction technique) using the *Inspect3D* (FEI) software.

The surface morphology and phase contrast were taken using an MFP-3D Stand Alone commercial atomic force microscopy system (Asylum Research, USA) in tapping/AC air topography operating mode. An Asyelec-01 cantilever with a resonant frequency of 120 kHz and a spring constant *k* = 2 N m^−1^ was used. The typical scanning areas were 2 × 2 µm, the scanning rate being 0.8 Hz. The topographic images were analyzed using the *Gwyddion* software (http://www.gwyddion.net is supported by the Department of Nanometrology, Czech Metrology Institute, Brno, Czech Republic).

## 3. Results and discussion   

**Figure 1:**
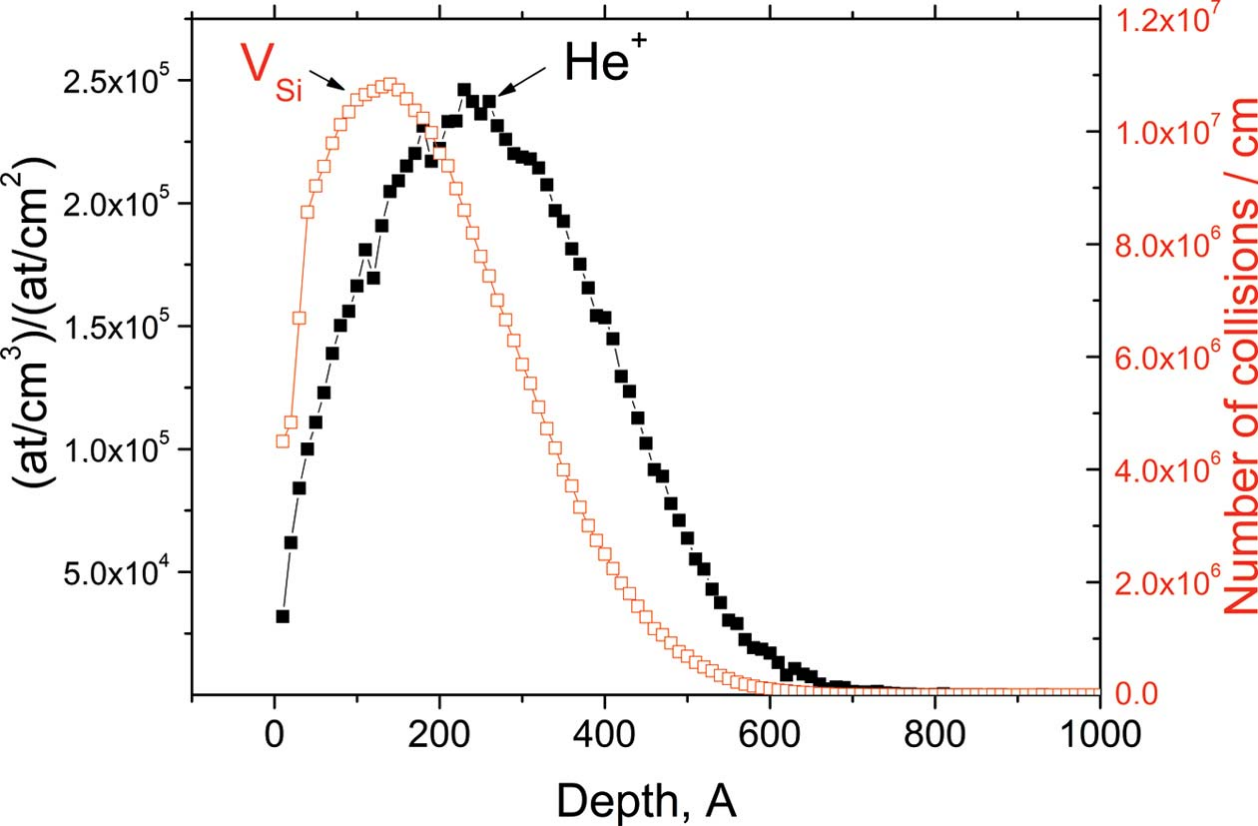
*SRIM* depth distributions of (filled squares) primary He^+^ ion and (open squares) Si vacancies (V_Si_). Ion projected length *R*_p_ ≃ 26 nm, number of vacancies per ion approximately 30.

In this study, the implantation conditions (energy and dose) were chosen such as to induce the formation of bubbles, *i.e*. gas-filled cavities. It is well known that, for He^+^ implantation doses of above 1 × 10^16^ cm^−2^, helium atoms agglomerate in the form of bubbles. A local helium concentration of ∼3 × 10^20^ cm^−3^ is required to trigger the formation of He-filled bubbles (Raineri *et al.*, 1995 ▸). The bubbles localize in a high vacancy (V_Si_) concentration region. The normalized depth profile of helium ions and the corresponding vacancy profile obtained by the *SRIM* code (http://www.srim.org) for He^+^ ion implantation at 2 keV with *D* = 5 × 10^17^ cm^−2^ are shown in Fig. 1 ▸. For 2 keV He^+^ ions, the projected range (*R*_p_) of He^+^ is approximately 26 nm. In comparison to the He^+^ ion distribution, the distribution of vacancies produced by implantation has its maximum closer to the surface (by ∼16 nm).

The fluence used in this experiment is far above the amorphization threshold, which is 1.5 × 10^16^ cm^−2^ according to the *SUSPRE* software (http://www.surrey.ac.uk/ati/ibc/research/modelling_simulation/suspre.htm). Therefore, one would expect amorphization of the damaged layer in the as-implanted state. The amorphous layer thickness as predicted by *SUSPRE* simulation is about 60 nm.

**Figure 2:**
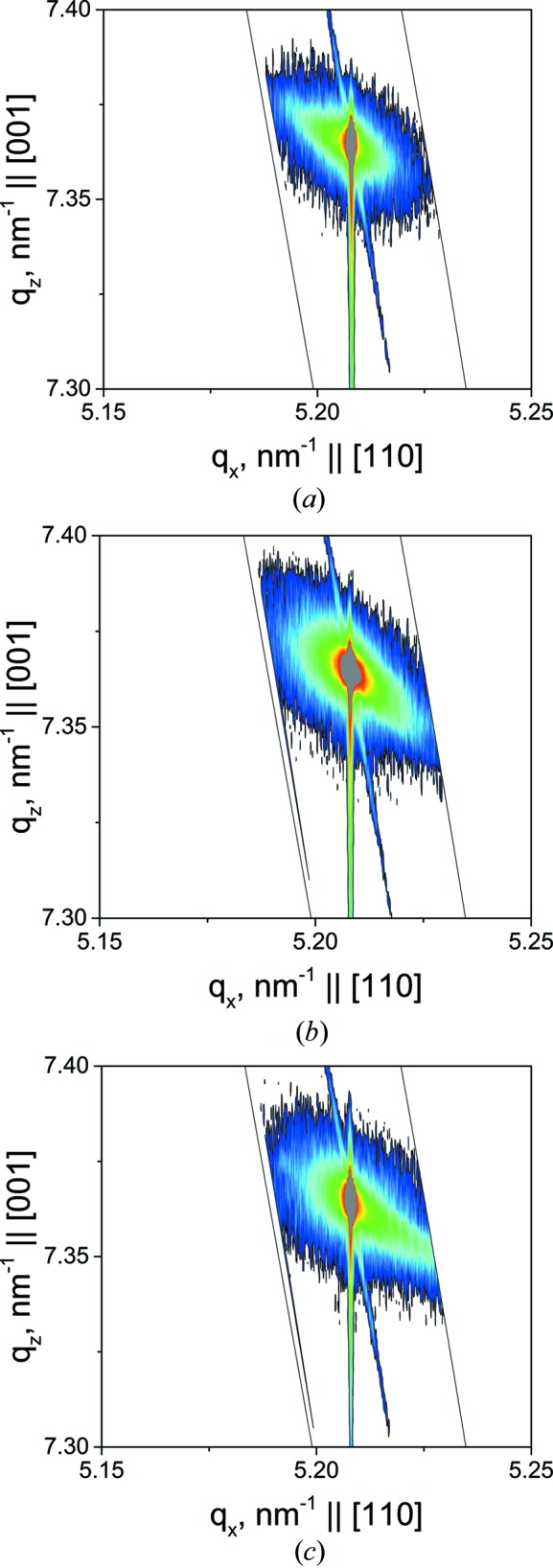
Si(224) RSMs measured for (*a*) as-implanted sample, (*b*) sample annealed at 853 K for 30 min and (*c*) sample annealed at 1073 K for 30 min.

Reciprocal space maps (RSMs) were taken near the Si(224) reciprocal lattice point (RLP) (Fig. 2 ▸). The Si(224) RLP is non-uniformly broadened perpendicular to the scattering vector **Q**(224), indicating the presence of a mosaic structure (slightly misoriented crystalline blocks) in as-implanted and annealed samples. Unlike Pivac *et al.* (2003 ▸), we did not observe any changes to the X-ray diffuse scattering (XRDS) intensity from radiation-induced interstitial-type defect clusters (rod-like defects and dislocation loops) in the annealed samples because of X-ray scattering on the mosaic blocks. The RSMs for all the samples demonstrate the presence of a tensile-strained crystalline layer, the layer and the matrix crystal lattice being completely coherent. This layer is a solid solution of radiation-induced point defects and small helium bubbles, which produce tensile strain in the layer. According to RSM analysis (Fig. 2 ▸), the crystal lattices of the damaged layer and the substrate remain coherent. This also means that the damaged layer is not fully amorphous.

Taking into account that one He^+^ ion generates up to 30 Frenkel pairs (vacancies V_Si_ and interstitial atoms Si_i_) and that Si_i_ are highly mobile even at room temperature, Si_i_ diffusion into the virgin substrate should be expected. This process also leads to the formation of a tensile-strained layer that can be revealed by analyzing the coherent part of the X-ray scattering.

**Figure 3:**
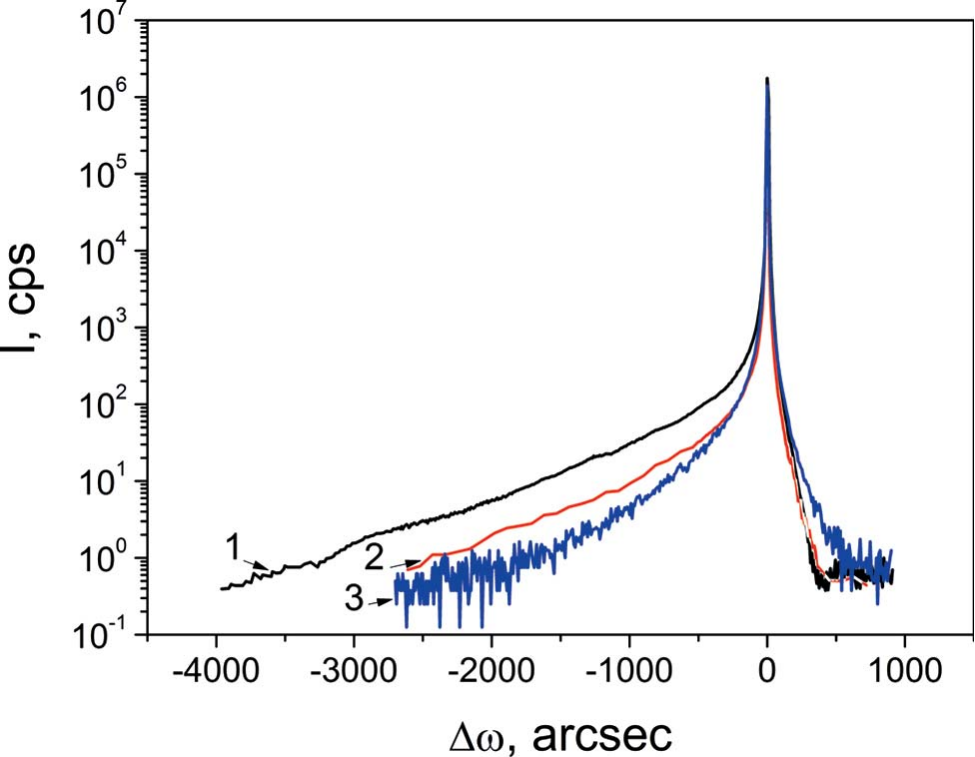
ω–2θ scans for Si(004) (coherent part of scattering): (1) as-implanted sample, (2) sample annealed at 853 K and (3) sample annealed at 1073 K.

RSM analysis (Shalimov *et al.*, 2007 ▸) was used to extract the coherent part of the scattering from the scattered intensity distribution around the Si(004) reciprocal lattice point (Fig. 3 ▸). The curves do not have any intensity fringes. Thus, accurate determination of the crystalline layer thickness is a difficult task even for the as-implanted sample. This fringe-free asymmetric shape of the diffraction curves suggests scattering at a crystalline layer with the tensile strain ∊_*zz*_ normal to the surface and decreasing monotonically into the depth of the substrate.

**Figure 4:**
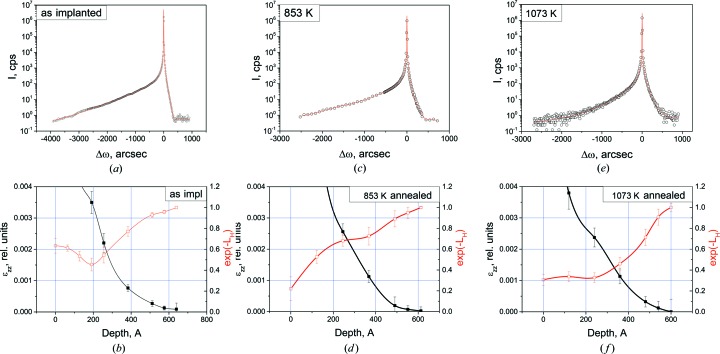
ω–2θ scans for Si(004) (coherent part of scattering) and best-fit curves (*a*), (*c*), (*e*) and strain ∊_*zz*_(*z*) and static Debye–Waller factor exp(−*L*_H_)(*z*) profiles (*b*), (*d*), (*f*): (*a*), (*b*) as-implanted, (*c*), (*d*) annealed at 853 K and (*e*), (*f*) annealed at 1073 K.

To get detailed information on the damage distribution in the crystalline Si layer (c-Si), we fit the ‘pure’ coherent part using an original procedure (Shcherbachev *et al.*, 2003 ▸), based on a genetic algorithm. The damage profiles which are described by the strain ∊_*zz*_(*z*) and static Debye–Waller factor exp(−*L*_H_)(*z*) profiles were obtained (Fig. 4 ▸). The parameter *L*_H_ is proportional to the mean-square displacement (Krivoglaz, 1996 ▸) and can be considered as a characteristic of point defect clustering degree. Diffraction curves were simulated in the framework of the dynamical X-ray scattering theory using the formalism suggested by Wie *et al.* (1986 ▸).

The strain gradually decreases in the depth of the c-Si layer. This is typical of an amorphized surface layer after heavy-dose low-energy implantation. Unfortunately, HRXRD provides no information on the thickness of the amorphous layer. The decrease of the exp(−*L*_H_) factor after heat treatment is due to growth of helium-filled bubbles with annealing temperature in the crystalline part of the damaged layer. At the same time, the shape of the strain profiles does not change noticeably, although high-temperature annealing is expected to reduce the number of radiation-induced defects. Thus, one can assume that helium-filled bubbles are the major source of strain in the c-Si layer. High-temperature annealing leads to He out-diffusion from small-size bubbles and the formation of nanovoids. The nanovoids are effective sinks for self-interstitials Si_i_. The reduction of Si_i_ supersaturation due to the presence of the nanovoids also hinders the formation of interstitial-type clusters (Giannazzo *et al.*, 2005 ▸).

**Figure 5:**
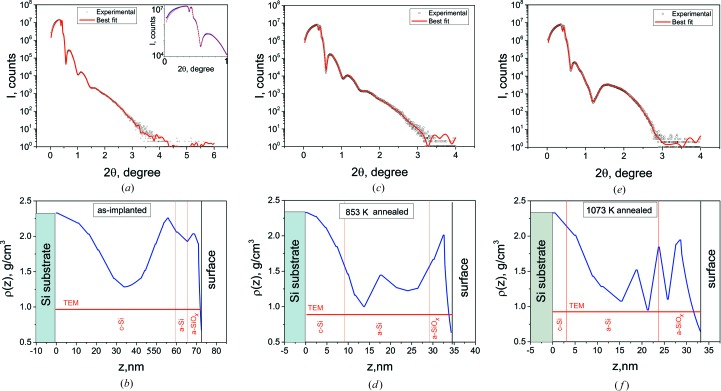
X-ray specular scattering and best-fit curves (*a*), (*c*), (*e*) and mass density profiles (*b*), (*d*), (*f*): (*a*), (*b*) as-implanted, (*c*), (*d*) annealed at 853 K and (*e*), (*f*) annealed at 1073 K. The inset in (*a*) shows part of the experimental curve in the vicinity of the critical angle. Information on layer thickness obtained by TEM is added for comparison.

Intense formation of bubbles even in the shallow buried layer changes the density of the material, which can be characterized by X-ray reflectometry. Taking into account that the estimated thickness of the damaged layer is about 100 nm, we performed measurements in a high-resolution arrangement. The specular scattering curves and the best-fit curves are shown in Fig. 5 ▸, together with the respective density profiles determined by the fitting procedure in *Leptos V7.0* (Bruker Corporation, USA) software. We used a subsurface layer model consisting of 12–14 linked lamellas with a linear density gradient. The features near the critical angle indicate the presence of a layer with a lower density than the Si matrix [see inset in Fig. 5 ▸(*a*)], located near the sample surface. This low-density subsurface layer remained even after annealing. The density profiles corresponding to the best-fit solution are found to consist of two low-density regions. It can be seen that the density changes nonmonotonically with depth (Figs. 5 ▸ *b*, 5 ▸ *d* and 5 ▸ *f*).

We consider the change of the density profile during thermal annealing in more detail. In the as-implanted state the density minimum is located at a depth of about 30–40 nm, which is slightly deeper than *R*_p_ determined by *SRIM*. One can assume that this minimum originates from the formation of helium-filled bubbles. It does not contradict the data specifying that the bubbles form in an as-implanted state for doses of above 5 × 10^16^ cm^−2^ (Raineri *et al.*, 1995 ▸). The minimum near the surface at a depth of about 10–15 nm can be associated with complexes of vacancies, which accumulate near the physical surface. These complexes can serve as traps for helium atoms. Helium is repelled by vacancies and forced to diffuse intensely during implantation. Instead, helium atoms are trapped by divacancies, stabilizing them and favoring their evolution to more complex He–V_Si_ clusters at temperatures of up to 673 K (Raineri *et al.*, 2000 ▸). According to the *SRIM* simulation (Fig. 1 ▸), the surface layer is supersaturated with V_Si_ and He atoms. Helium-filled bubbles form even in the as-implanted state [see the TEM tomography image below, Fig. 7(*b*)]. Thus, the density is more likely to be determined by helium-filled bubbles than by sole vacancy complexes. In the amorphous layer, the bubbles are larger than those in the crystalline layer. This caused singularities in the density distribution in Fig. 5 ▸(*b*).

**Figure 6:**
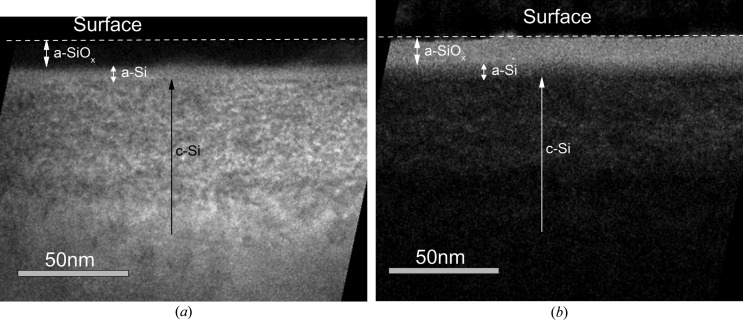
Energy-filtered TEM images of (*a*) Si and (*b*) SiO_*x*_ layers.

The formation of three regions in the damaged layer, *i.e.* (i) an amorphous SiO_*x*_ layer (a-SiO_*x*_) on the surface, (ii) an amorphous Si layer (a-Si) and (iii) a heavily damaged crystalline layer (c-Si), was revealed by TEM (Fig. 6 ▸). A possible explanation of the formation of an SiO_*x*_ layer with a greater thickness than that of the native oxide is that helium plasma irradiation leads to chemical activation of the Si surface. Oxidation occurs during air filling of the chamber (backfill) where PIII is performed. The thickness of the oxide layer does not change after heat treatment. According to HRXRR data (Fig. 5 ▸ *b*), the c-Si layer has a low density. At the same time, according to HRXRD (Fig. 4 ▸ *a*), this layer is tensile strained. The major radiation-induced defects in this layer are Si_i_, and they could be the source of the tensile strain. However, intrinsic interstitials increase the density. Thus, the source of the strain is the helium-filled bubbles.

This three-layered structure remains after annealing. The thicknesses of these layers in as-implanted and annealed samples are presented in Table 1 ▸. For comparison with HRXRR data, TEM data on the layer thicknesses are shown in Fig. 5 ▸. The comparison shows a discrepancy between the c-Si layer thickness results obtained in HRXRR and TEM experiments.

One reason is that the thickness of the disturbed crystalline Si layer (*t*_def-Si_) was determined from dark-field TEM images. The contrast of the TEM image of this layer has a diffraction nature. At the same time, the electron density contrast is much weaker. Thus, the TEM thickness of the c-Si layer must be greater than the HRXRR one. Another reason is that the HRXRR density profile is obtained by fitting a rather complex interference pattern where the main contribution comes from areas with a sharp density gradient. The shape of the curve is less sensitive to layers where the density changes slightly with depth.

**Figure 7:**
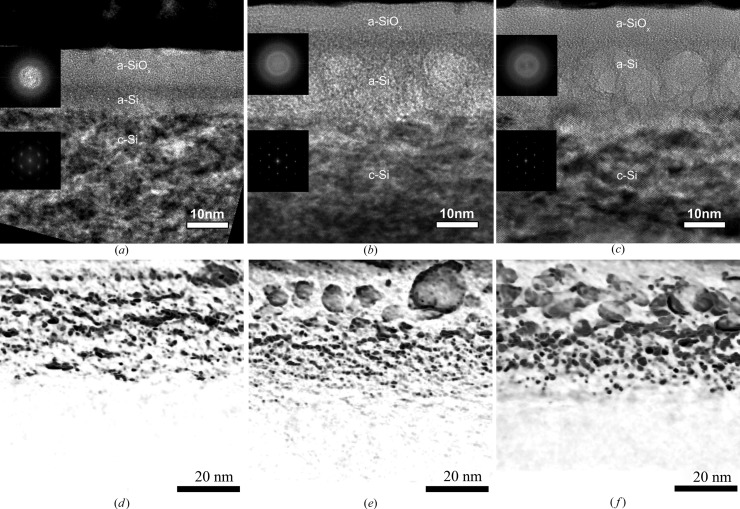
(*a*), (*b*), (*c*) TEM and (*d*), (*e*), (*f*) inverted tomography TEM images: (*a*), (*d*) as-implanted, (*b*), (*e*) 853 K and (*c*), (*f*) 1073 K. The oxide layer is not shown in the tomography images. Large-size helium-filled bubbles are located in the amorphous Si layer. ‘Small’ bubbles are located inside the distorted crystalline Si layer.

After annealing at 853 K, which facilitates the growth of helium-filled bubbles, the thickness of the layer with the changed density decreases approximately twofold (Fig. 5 ▸ *d*). Two minima at a depth of about 23 and 10 nm are clearly visible in the density profile. According to TEM (Figs. 7 ▸ *a* and 7 ▸ *d*), in the amorphous region large (∼10 nm) bubbles are located closer to the surface and small ones (∼4 nm) are observed at a greater depth. The ‘tail’ of the distribution of the small bubbles extends into the heavily damaged crystalline area c-Si. It should be noted that, since the thickness of the c-Si layer decreases to 50 nm after 853 K annealing (see Table 1 ▸), a decrease in the integral intensity of scattering at the layer (Fig. 3 ▸) is caused by enlargement of the bubbles [which indirectly confirms the decrease of the exp(−*L*_H_) factor in Fig. 4 ▸(*b*)].

**Figure 8:**
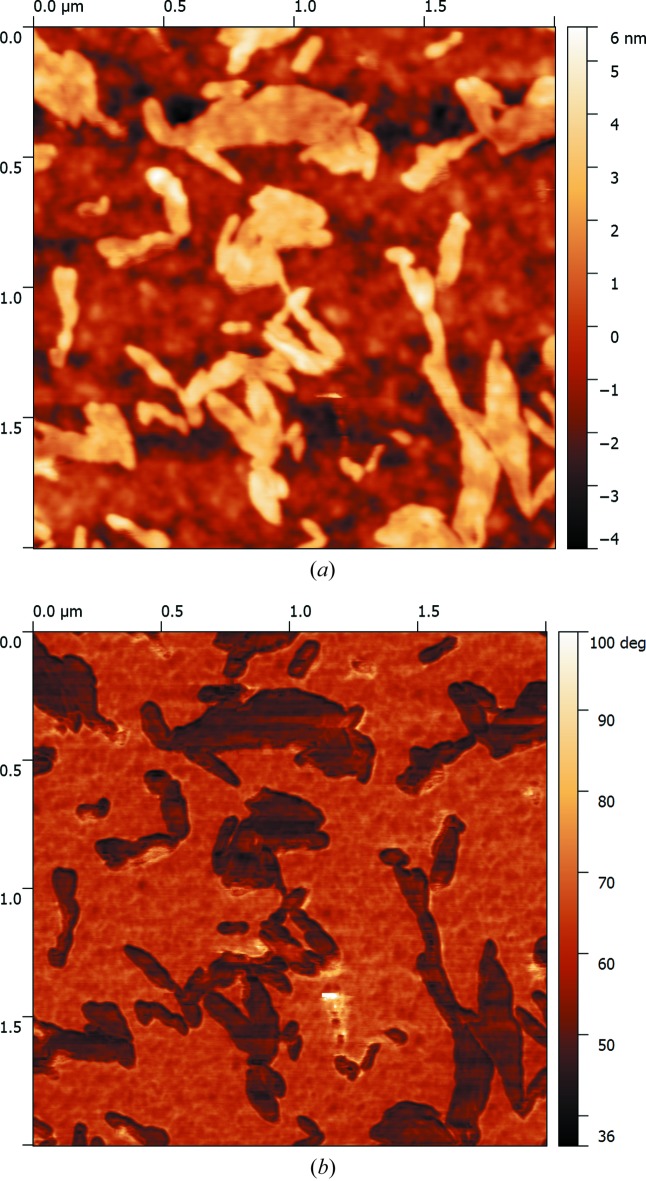
AFM topography (*a*) and phase contrast (*b*) images for the sample annealed at 1073 K.

After annealing at 1073 K, significant changes in the shape of the density profile, especially near the surface (Fig. 5 ▸ *f*), are observed. An AFM topography image of the surface (Fig. 8 ▸ *a*) of this sample shows a well developed relief. The value of the mean-square roughness increased from 0.44 nm for the as-implanted and the 853 K annealed samples to 1.50 nm for the sample after 1073 K annealing. This may result from blistering and exfoliation effects. A phase image (Fig. 8 ▸ *b*) shows that the exfoliated layer differs chemically from the underlying one. This exfoliated layer with a thickness of 9 ± 1 nm is silicon oxide (SiO_*x*_). The oxide layer has been shown to be an effective barrier to helium diffusion towards the surface during subsequent annealing (Liu *et al.*, 2008 ▸). Thus, the blocked He atoms diffuse inward and are recaptured by bubbles, leading to the formation of over-pressurized bubbles in the damage region close to the original interface.

According to the TEM analysis (Fig. 7 ▸ and Table 1 ▸), the thickness of the amorphous layer a-Si where large (∼10 nm) bubbles are located increases. A decrease in the thickness of the disturbed crystal layer c-Si is probably caused by out-diffusion of He atoms, a decrease in the number of small bubbles, and their transformation either to larger-size bubbles or to nanopores which, in turn, are traps for Si_i_. All these factors reduce the tensile strain. The reduction in the thickness and strain of the c-Si layer leads to a decrease in the integral scattering intensity at the c-Si layer (Fig. 3 ▸).

## 4. Conclusions   

An application of a combination of complementary methods for a comprehensive study of the structural properties of Si(001) subsurface layers after high-dose low-energy (2 keV) He^+^ PIII is presented. The layers formed by PIII have a complex structure with a variety of parameters that cannot be characterized using only one method. For this reason, high-resolution X-ray diffraction and reflectivity, transmission electron microscopy, and atomic force microscopy experiments were performed. The possibility of using the conventional X-ray method to extract thickness, strain and amorphization profiles and their transformation during subsequent thermal annealing is demonstrated.

It was found that, in the He^+^ PIII Cz-Si(001) substrate under the present experimental conditions, a three-layer structure (an amorphous a-SiO_*x*_ layer at the surface, an amorphous a-Si layer and a heavily damaged tensile-strained crystalline c-Si layer) is formed, which remains after annealing at 853 and 1073 K. The structure was revealed by HRXRR and confirmed by TEM. The density depth profiles reconstructed from the specular HRXRR curves are not uniform throughout the depth of the damaged layer, and a sharp interface with the Si substrate is absent.

Helium-filled bubbles are revealed in the as-implanted sample. They grow after annealing. The characteristic bubble size is estimated from TEM tomography images to be 4–10 nm. Large-size helium-filled bubbles are located in the amorphous a-Si layer. Small-size bubbles are revealed inside the crystalline c-Si layer at depths of about *R*_p_. These bubbles are a major source of tensile strain in the c-Si layer.

High-temperature annealing (1073 K) leads to the formation of a well developed relief on the surface caused by blistering and exfoliation, which is confirmed by AFM. HRXRD confirms the presence of slightly misoriented crystalline blocks in the as-implanted and annealed samples. In contrast to high-energy He^+^ ion implantation, HRXRD did not reveal any XRDS from radiation-induced interstitial-type defects that form clusters (rod-like defects and dislocation loops) in the annealed samples.

In conclusion, the results of this study demonstrate how a combination of the HRXRD and HRXRR techniques on a laboratory diffractometer constitutes a fundamental tool in the study of thin complex implanted structures. However, to have a comprehensive model of microstructure and its evolution, X-ray scattering should be used in combination with other complementary methods. This combination proved to be a powerful tool for complete structural diagnostics of nanoscale He^+^ ion implanted Si layers, especially for low-energy implantation.

## Figures and Tables

**Table 1:** Thickness of amorphous SiO_*x*_ (*t*_a-SiO_*x*__), amorphous Si (*t*_a-Si_) and damaged strained crystalline Si (*t*_def-Si_) determined from TEM images

Sample	*t*_a-SiO_*x*__ (nm)	*t*_a-Si_ (nm)	*t*_def-Si_ (nm)
As implanted	8 ± 1	5 ± 1	65 ± 5
853 K	7 ± 2	20 ± 2	50 ± 5
1073 K	9 ± 3	23 ± 2	45 ± 5
